# The Women's Hospital for Children, Harrow Road

**Published:** 1914-01-31

**Authors:** 


### THE WOMEN'S HOSPITAL FOR CHILDREN, HARROW ROAD.

### An Institution Attempting Too Much.

This institution began as an out-patient clinic in 1912, and in December 1913 a small in-patient department was opened in addition. As the name implies, it is managed and staffed by women. It is housed in three adjacent workmen's cottages at 688 Harrow Road, near where Ladbroke Grove debouches into that thoroughfare. The neighbourhood contains a great deal of tenement and similar property, and a large proportion of the inhabitants belong to what may be termed the hospital classes; those, that is, who are not sufficiently indigent to obtain medical aid through the Poor Law, but yet are unable to pay any but the most minute fees for private medical attendance. Two of the cottages aforesaid constitute the out-patient department, and the remaining one is devoted to in-patients.

### The Out-Patient Clinic.

The out-patient department has already treated over 7,000 patients since it started about a year and a half ago. Monday is the busiest day; one hundred to one hundred and ten is about the usual attendance on that afternoon, and nearly as many are seen on the other out-patient days (five afternoons a week in all).

As at the other children's hospitals, waiting accommodation for the children involves provision for about twice the actual number of patients, because the latter are accompanied by parents or guardians. There is a yard at the back of the premises where perambulators are stored; but there has been trouble with thefts, and improved arrangements will be necessary to put a stop to this annoyance. When the rush is at its height, there is not nearly sufficient accommodation for the crowd of mothers and children, and many have to take up their stand in the yard. At the time of our visit this inconvenience was not in evidence, as there were empty chairs to be seen in the waiting-rooms; but at that time the total number of parents and children in waiting cannot have exceeded forty or fifty.

The consulting rooms are small; but this is not a serious handicap in a children's out-patient clinic. The ophthalmic room seemed to be best equipped and adapted for its purpose; but the medical equipment in general of the rooms is restricted and capable of much improvement. One of the consulting rooms, a very small one, is used for elementary electro-therapy, and also as an operating room, both for in-patients and out-patients. It has not been properly adapted for this latter purpose, and only the most trivial operations could be safely attempted in it. The medical officers are stated to be contemplating major surgery in the near future: at present, cases requiring it are referred elsewhere. It would be quite unjustifiable to do any major surgery in this hospital at all; and even about minor surgery one feels misgivings.

One of the kitchens of the original cottages is used as an isolation room for infectious cases, pending their removal to an infectious diseases hospital. It contains a fireplace and two wooden chairs—nothing else whatever.

The dispensary is a corner of a small room, fitted with a counter and drawers, and a few shelves and bottles. So far the medical officers have dispensed their own prescriptions, a curious system to which they are not unnaturally beginning to object. The appointment of a dispenser is contemplated by the committee.

The nursing staff of the out-patient clinic consists of one trained nurse, and one assistant nurse who is not fully trained.

Parents are required to pay sixpence a week towards the cost of the medicine, treatment, and so forth, the children attending twice a week as a general rule. The revenue from this source is estimated at about £5 a week, or even more. Those who can prove inability to afford this fee are excused payment. The secretary acts also as almoner. Her office is a tiny room, almost filled with children's toys and parcels of various kinds; and the committee room is similarly encumbered.

### The In-Patient Department.

The ward where in-patients are received is on the first floor, and accommodates seven children, only one of whom may be under two years of age. The space available in this room is ingeniously utilised, and it is only because the ward furniture is scanty that overcrowding does not exist. The children look happy and well cared for; apparently trivial cases are admitted such as elsewhere would be treated as out-patients. The beds, bedding, and linen are reasonably good; but medical and surgical accessories are deficient.

The bath-room is a minute apartment on the same floor, and it has to serve not only for the patients, but also for the nurses, a thoroughly vicious arrangement. More than that, it forms the ward kitchen as well! The bath looks hardly large enough for an adult person to get into. Hot water is supplied by a geyser, not a good plan to adopt in a children's hospital. In a corner of the room is a gas-cooker. A board is kept over the top of the bath, for use as a table. As the whole space is about eight feet by six, it will be realised that the bathing accommodation for the in-patient department is primitive and inadequate. A portable tin hip-bath is used for infants. The sanitary arrangements are what might be expected in a cottage, and are rudimentary to a degree.

Downstairs are a kitchen and scullery, and a nurses' room, which is used both as a dining-room and sitting-room. The furniture looks comfortable, but the drawback to the room is a fireproof door opening, through what was originally the party-wall, into the out-patient department. This is the only communication inside the building between the two departments, and it cannot make for peace and quiet in the little sitting-room.

The kitchen is of ordinary size for this type of property; and the supply of pots, pans, and crockery looks insufficient for the needs of the establishment. In a scullery opening out of it some washing is done, such as baby napkins and muslin curtains; but the bed-linen, underwear, and so forth is sent out to be washed. This department is worked by two women who live out. One of them acts as cook. The children's tea was ready for serving at the time of our visit, and looked excellent.

There are three fully trained nurses, each of whom has a separate bed-room, exceedingly tiny. They work in three shifts, taking nine hours' consecutive duty, without any time off during duty hours. At present there is neither matron nor resident medical officer.

### Finance.

No charge is made to the parents of in-patients; but the committee are about to take this matter under consideration. About £4,000 is in hand for a building fund; but the committee are not bound to the present site, and may possibly, when the time comes, choose some other in the neighbourhood. Until lately the income of the institution has been subscribed privately. But recently the public has been appealed to for subscriptions, and a good deal of local support has been obtained. The trade unionists of the district voluntarily held a "demonstration" in 1913, which brought in £45; and have decided to repeat this effort annually. A "Pound Day" was held about the end of last year, and realised £100 in cash, besides many gifts in kind.

### Conclusions.

The out-patient clinic is evidently popular in the district. There is no other clinic quite near; but Paddington Green Children's Hospital is only about a mile and a half distant, and easily accessible by trams and motor-buses. Notwithstanding the restricted accommodation, good work is done, and a great deal of it; but undoubtedly a properly equipped clinic, though more expensive, would reach a higher level of excellence. The clinic may fairly claim to have justified its formation, and to deserve further support with a view to increasing its efficiency. It would be prudent to restrict the number of cases seen to fifty or sixty an afternoon; one hundred is far too many for the resources of the clinic at present.

The in-patient department has, perhaps, hardly had time to settle down into working order. It is obvious, however, that the building is wholly improper for an in-patient department, and that it was a serious blunder ever to start one in it. It can be argued that the children admitted have a far better chance of recovery than in their own homes in the adjacent slums. But they cannot enjoy anything like first-class care, such as other voluntary hospitals afford. If it can be shown that Paddington Green Hospital and the nearest general hospitals are habitually turning children away for lack of beds, some slight degree of justification may exist for this in-patient department; otherwise, to take children in here is a grave scandal. The committee have doubtless meant well, but they would have done much better for their patients by improving the out-patient clinic with the money now being spent on in-patients, leaving serious cases to be transferred to other hospitals.

The resources of the institution appear to be so strained by the programme now being undertaken, that economy has to be pressed to the extreme of parsimony. There is a general air of skimped furnishing and accessories, which shows that too much is being attempted. Many hospitals, now of established fame, have started in as humble a home and as unostentatiously as this one; but sanitary conditions and hospital environment which were tolerated a century ago can no longer be excused today. It was not in the least necessary to be in such a hurry over starting an in-patient department, which could well have been deferred until the building fund enabled a proper hospital to be erected on a suitable site. Assuming that public support for this institution continues to increase, it would be wise of the committee to undertake no further liabilities and to permit no further expansion until the income of the charity has allowed many now badly needed improvements to be put in hand and carried out.

